# Supplementary material for: Diel population and functional synchrony of microbial communities on coral reefs
Source: Nat Commun. 2019 Apr 12;10:1691. doi: 10.1038/s41467-019-09419-z (PMC6461649; doi:10.1038/s41467-019-09419-z)
Supplement: Supplementary file 1 — Supplementary Information [file 41467_2019_9419_MOESM1_ESM.pdf]

Supplementary Information

Supplementary Methods

Sample sites and benthic cover. Field studies were conducted across four islands in the Southern Line Island archipelago (southern portion) spanning 4°01'S to 11°26'S from October 18 to November 6, 2013. Mean daily PAR at 10m depth on the forereef measured of 312 +/- 214 μmol photons m^{-1} s^{-1} (LI-COR, Inc., www.licor.com) and water temperature ranged from 28.1 +/- 0.5 ^{o}C. The percent cover of benthic corals and algae was estimated in each tent mesocosm using photoquads and the program photogrid as described in Smith et al., (1).

In situ *reef collections.* Collapsible benthic isolation tents (cBITs), referred to in text as benthic chambers, were used to assess effects of specific benthic communities *in situ*. The triangular pyramids, which were developed and built at the Smith and Rohwer laboratories, primarily consist of three transparent polycarbonate side panels joined by flexible polyvinyl chloride strips connected by stainless steel cables (as described in Haas et al., (2)). Benthic chambers were deployed at 10 m on the fore reef habitat on all four southern Line Islands described in this study. All benthic chambers were equipped with autonomous multiprobe (MANTA2 Eureka Water Probes, www.waterprobes.com) that monitored temperature (precision 0.01°C), DO (precision 0.01 mg l-1, accuracy ±1%, automatic temperature and pressure compensated and salinity corrected), pH and conductivity (accuracy ±1%, automatic temperature compensated) at 15 min intervals.

The rate of water exchange in the benthic chambers was calculated using dilution rates of Fluorescein dye. Fluorescein dye was injected into control chambers on Malden, Millennium, and Starbuck and the concentrations measured using a multiprobe sensor with a fluorometer (precision 0.01ppb; Manta2, Eureka Water Probes, TX, USA). Chamber flushing rates, F (liters min^{-1}) were calculated from the dilution rate, D (slope, log10ppb min^{-1} divided by the initial concentration, log10ppb) multiplied by chamber volume (100 liters). Mean flushing rates were 5.04, 5.48, and 2.52 liters min^{-1} for Malden (n=3), Millennium (n=4), and Starbuck (n=5), respectively (Supplementary Figure 1).

Sequence library preparation and bioinformatics. Nucleic acids were extracted from microbial communities collected on Sterivex filters using a modified protocol of the Nucleospin Tissue Kit (Machery-Nagel, Santa Clara, USA) as described previously by Kelly et al., (3). DNA for each sample was normalized to 0.2ng/ul and libraries were built using Nextera XT (Illumina, San Diego, USA). Metagenomic libraries were sequenced on the MiSeq2 using the 600 cycle PE sequencing reagent kit (Illumina, San Diego, USA). The shotgun sequence libraries were post-processed using Prinseq (4) to remove low quality reads (ambiguous bases, low complexity, short read length, replicates). Sequence reads were compared to the SEED database for metabolic and taxonomic assignments using SUPERFOCUS (5), which aligns sequence similarities using RAPSearch2 (6) and performs a 98% clustering of the proteins in the database to reduce computational taxation. Metagenomic reads aligned against the SEED database to get the functional annotation were subsequently extracted to identify the taxa that are encoding the respective protein coding genes from NCBI using Taxonkit (http://bioinf.shenwei.me/taxonkit/). For putative 16S rRNA gene sequences, alignment, classification, sequence distance calculation, OTU clustering, phylogenetic tree construction and calculation of among-sample phylogenetic distances were done using the software package mothur (7) following previously published bioinformatics pipelines (8). Additional phylogenetics were conducted using the SINA multiple

sequence alignment algorithm (9) and the RaxML maximum likelihood phylogeny reconstruction algorithm (10).

Supplementary References

1. Smith JE, *et al.* Re-evaluating the health of coral reef communities: baselines and evidence for human impacts across the central Pacific. *Proceedings of the Royal Society B* 283: rspb20151985 (2016).

2. Haas AF, *et al.* Influence of coral and algal exudates on microbially mediated reef metabolism. *PeerJ* 1:e106 (2013).

3. Kelly LW, *et al.* Local genomic adaptation of coral reef-associated microbiomes to gradients of natural variability and anthropogenic stressors. *P Natl Acad Sci USA* 111(28):10227-10232 (2014).

4. Schmieder R & Edwards R. Quality control and preprocessing of metagenomic datasets. *Bioinformatics* 27(6):863-864 (2011).

5. Silva GGZ, Green KT, Dutilh BE, & Edwards RA. SUPER-FOCUS: a tool for agile functional analysis of shotgun metagenomic data. *Bioinformatics* 32(3):354-361 (2016).

6. Zhao Y, Tang H, & Ye Y. RAPSearch2: a fast and memory-efficient protein similarity search tool for next-generation sequencing data. *Bioinformatics* 28(1):125-126 (2012).

7. Schloss PD, *et al.* Introducing mothur: Open-Source, Platform-Independent, Community-Supported Software for Describing and Comparing Microbial Communities. *Appl Environ Microb* 75(23):7537-7541 (2009).

8. Nelson CE & Carlson CA. Tracking differential incorporation of dissolved organic carbon types among diverse lineages of Sargasso Sea bacterioplankton. *Environ Microbiol* 14(6):1500-1516 (2012).

9. Pruesse E1, Peplies J, Glöckner FO. SINA: accurate high-throughput multiple sequence alignment of ribosomal RNA genes. *Bioinformatics* 28(14):1823-1829 (2012).

10. Stamatakis A. RAxML version 8: a tool for phylogenetic analysis and post-analysis of large phylogenies. *Bioinformatics.* 30(9):1312-1313 (2014).

11. Benjamini Y & Hochberg Y. Controlling the False Discovery Rate - a Practical and Powerful Approach to Multiple Testing. J Roy Stat Soc B Met 57(1):289-300 (1995).

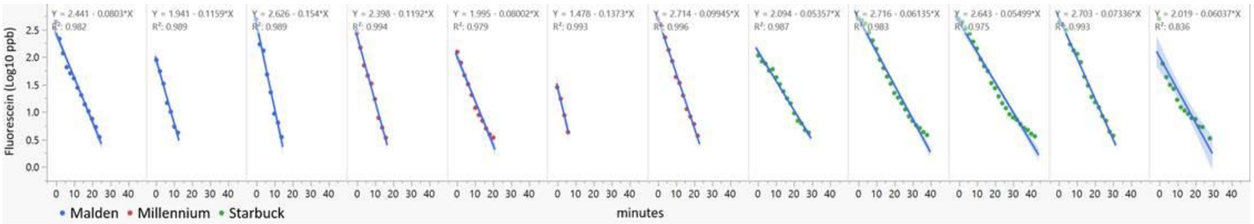

Supplementary Figure 1. Measurement of benthic chamber flushing rates. Estimates of water exchange in the benthic chambers were calculated using Fluorescein dye dilution rates. Dilution of Fluorescein dye was measured in control chambers on Malden, Millennium, and Starbuck using a multiprobe sensor with a fluorometer (Manta2, Eureka Water Probes, TX, USA). Chamber flushing rates, F (liters min^{-1}) were calculated from the dilution rate, D (slope, log10ppb min^{-1} divided by the initial concentration, log10ppb) multiplied by chamber volume (100 liters). Mean flushing rates were 5.04, 5.48, and 2.52 liters min^{-1} for Malden, Millennium, and Starbuck, respectively. Therefore, the turnover rate or *residence time* of seawater within the benthic chambers is estimated to be between 20 and 40 minutes.

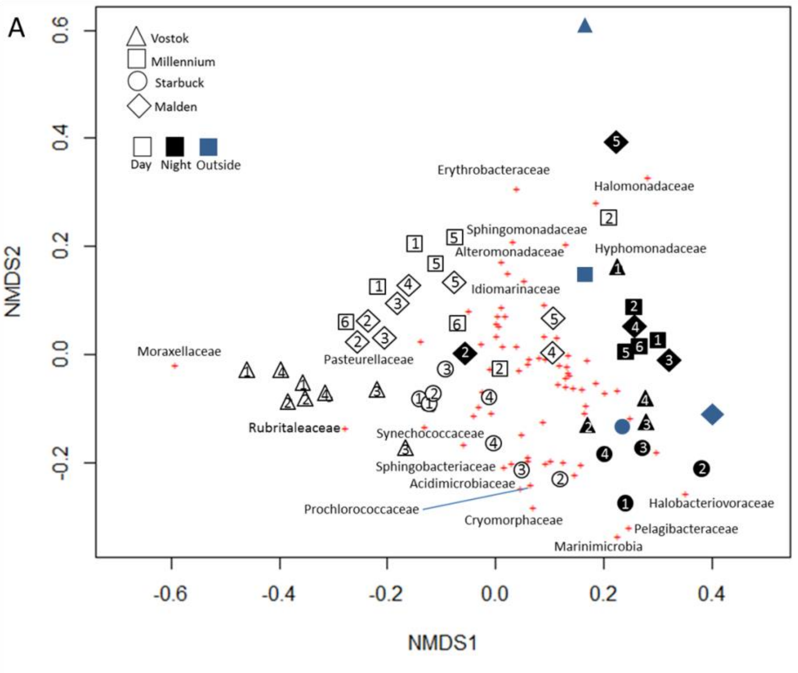

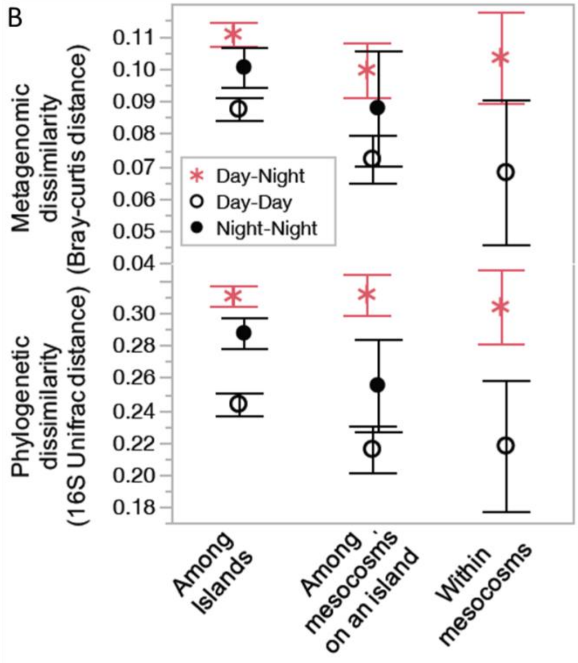

C

	F –stat	R^2	FDR Adj-p
Day (t0) – Day (t24)	*0.7109*	*0.0204*	*0.570*
Night (t12) – Outside (t12)	*0.9005*	*0.0430*	*0.570*
Day (t0) – Night (t12)	*8.1350*	*0.1930*	*0.003*
Day (t0) – Outside (t12)	*5.5530*	*0.2173*	*0.003*
Day (t24) – Night (t12)	*5.0342*	*0.1289*	*0.008*
Day (t24) – Outside (t12)	*3.6035*	*0.1526*	*0.024*

Supplementary Figure 2: Day-night community dissimilarity of reef microbes. (A) Nonmetric Multidimensional scaling (NMDS, stress = 0.13) of the metagenomic taxonomic composition of the reef microbial communities (Family level). Pairwise comparisons are grouped according to time of day (symbols) and location (categories across the x-axis); "Outside" refers to nighttime samples collected from the ambient reef, while T0 samples are considered daytime samples collected from the ambient reef. (B) Comparison of pairwise microbial community distances of protein coding genes (metagenomic dissimilarity) and species composition (phylogenetic dissimilarity) among sample sets; error bars depict the 95% confidence interval of the mean. (C) PERMANOVA tests (package *adonis* in R) of pairwise community dissimilarity showing that daytime ambient (T0) and tent (T24) communities do not differ ($p = 0.57$), nighttime ambient (outside) and tent (T12) communities do not differ ($p = 0.57$), but all other pairwise comparisons are significant ($p < 0.05$). Including Island in the full model or either daytime or nighttime data subsets always yields a significant Island effect and a nonsignificant interaction term, clarifying that diel patterning does not differ among islands. The false discovery rate was controlled by adjusting p-values according to Benjamini and Hochberg 1995 (11).

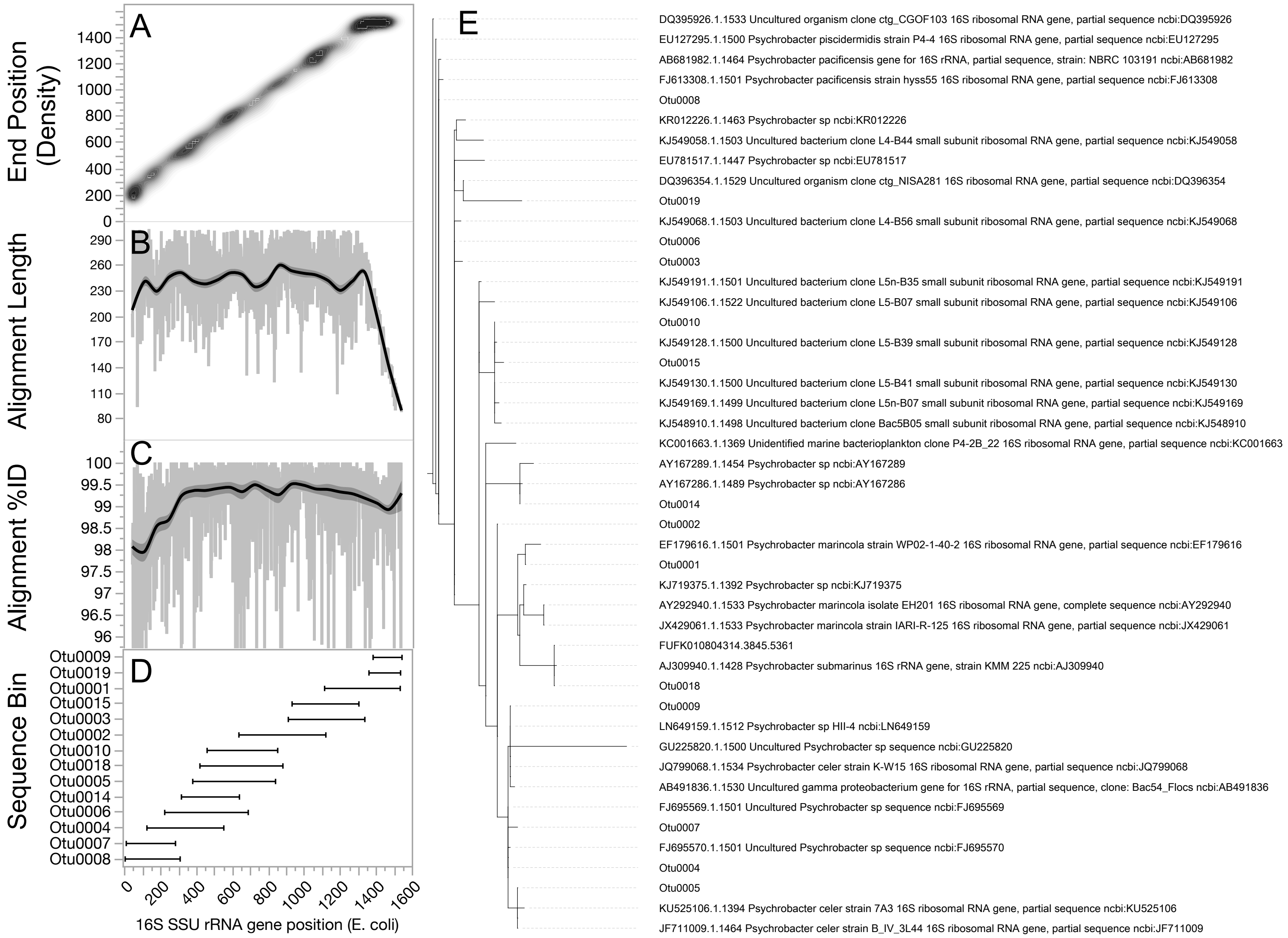

Supplementary Figure 3. Alignment of shotgun metagenomic 16S rRNA coding sequences assigned to OTUs classified as Psychrobacter by SINA alignment to the SILVA database. Distribution density (A), alignment length (B) and alignment identity (C) of reads clustered into positional OTUs (D) and consensus sequences visualized in a maximum likelihood phylogeny (E) with nearest neighbors from SILVA v132 RefNR and built by RaxML using the GTR model.

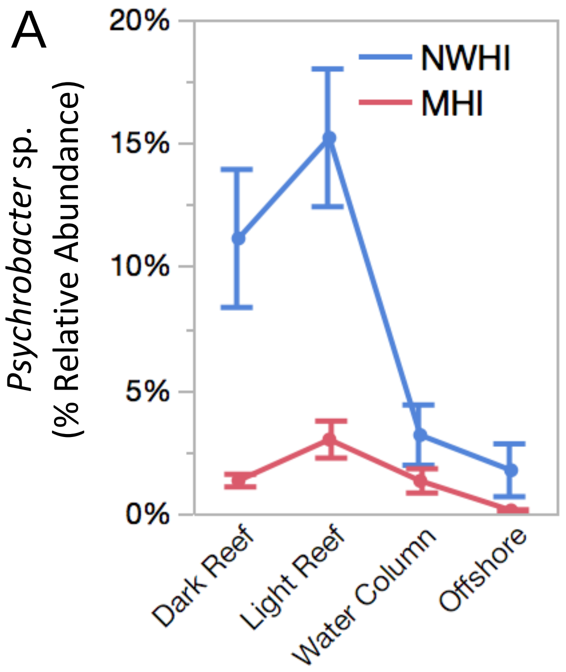

B

Island	Year Collected	% RA Moraxellaceae Min	- Max
Flint	2009	1.22	33.89
Vostok	2009	4.22	4.22
Millennium	2009	1.99	3.54
Starbuck	2009	1.31	1.76
Malden	2009	7.11	7.36
Jarvis	2010	0.32	17.28
Kiritimati	2010	0.62	0.67
Tabuaeran	2010	5.11	11.00
Teraina	2010	3.68	39.93
Palmyra	2010	0.55	0.55
Kingman	2010	0.79	0.79

Supplementary Figure 4: Prevalence of *Psychrobacter* spp. on 22 Pacific islands. (A) *Psychrobacter* sp. abundance (based on 16S rRNA gene amplicon libraries) in the Main and North-west Hawaiian Islands collected in 2016. (B) Moraxellaceae Family abundance from metagenomic libraries on 11 Line Island collected in 2009 and 2010 published in Kelly et al., 2014 (3). RA, relative abundance.

Supplementary Table 1 | Metagenomic library details and benthic cover for reef sites

			Metagenomic sequences				Benthic coverage				
Location	Tent	Time Point	Total QC Reads	Mean Length (bp)	Signif. Protein assign.	SSU rDNA aligns.	Stony coral	CCA	Calc macr algae	Flesh macr algae	Turf algae
Vostok -10.0609, -152.309	1	0	112419	226	43387	261	78.4	16.2	2.7	0.0	2.7
	1	12	160942	236	68245	361					
	1	24	289495	223	109807	567					
	2	0	551521	255	212725	1023	89.4	6.4	2.1	0.0	2.1
	2	12	261171	205	95229	470					
	2	24	216358	215	74548	462					
	3	0	145292	235	48379	263	80.9	14.9	2.1	2.1	0.0
	3	12	507301	235	197283	923					
	3	24	91486	220	34418	225					
	4	0	267200	236	105523	470	59.1	38.6	2.3	0.0	0.0
	4	12	601378	232	239285	894					
	4	24	1059949	245	424904	1795					
Millennium -9.95080, -150.215	1	0	216679	218	86649	396	45.7	0.0	37.0	0.0	17.4
	1	12	328081	229	117653	384					
	1	24	376630	162	100241	449					
	2	0	50094	237	21617	103	55.3	8.5	36.2	0.0	0.0
	2	12	238917	237	91397	303					
	2	24	227765	208	67977	295					
	5	0	434091	224	173168	658	25.5	6.4	40.4	4.3	21.3
	5	12	339092	207	109184	292					
	5	24	377883	175	123651	496					
	6	0	553261	238	212044	888	84.4	13.3	2.2	0.0	0.0
	6	12	437404	195	140268	487					
	6	24	170765	212	65008	318					
Starbuck -5.62891, -155.925	1	0	562775	231	218129	872	15.9	27.3	50.0	0.0	6.8
	1	12	397508	194	117393	467					
	1	24	374457	231	141614	635					
	2	0	582724	231	206118	914	0.0	15.2	82.6	0.0	2.2
	2	12	575240	219	169406	635					
	2	24	381406	229	119125	500					
	3	0	524438	229	166269	644	68.1	25.5	2.1	0.0	4.3
	3	12	588108	230	197717	643					
	3	24	389186	232	132111	546					
	4	0	418233	194	300986	1254	0.0	22.0	78.0	0.0	0.0
	4	12	326790	222	147150	496					
	4	24	959770	218	111198	534					
Malden -4.01407, -154.973	2	0	81666	257	34687	151	50.0	28.3	2.2	2.2	17.4
	2	12	246420	213	90704	405					
	2	24	207829	248	83601	415					
	3	0	215998	249	81969	430	91.8	8.2	0.0	0.0	0.0
	3	12	210712	204	81026	307					
	3	24	295866	210	116751	525					
	4	0	175898	274	64945	226	35.7	28.6	0.0	14.3	21.4
	4	12	229756	246	92219	345					
	4	24	198693	188	70448	338					
	5	0	280773	255	92874	296	41.7	20.8	0.0	2.1	35.4
	5	12	191105	263	90746	407					
	5	24	364154	219	141204	562					
**Total**	**16**	**3**	**16.8 mil**	**225**	**6.03 mil**	**25330**	**51.4**	**17.5**	**21.2**	**1.56**	**8.19**

QC, quality control; bp, base pairs; signif., significant; assign, assignments; align., alignment; CCA, crustose coralline algae; Flesh macr, fleshy macro-, Calc macr, calcified macro-; mil, million. Total represents sum or mean, where appropriate.

Supplementary Table 2 | Microbial taxa enriched during the day and night. Diel enrichment of Family level taxons (metagenomic sequence similarities to the SEED database) are shown for "Common", "Semi-common", and "Rare" taxa (maximum abundance >5%, 0.5% - 5%, and <0.5%, respectively). Significant enrichment (t-test, two-tailed, $p < 0.05$) and false discovery rate corrected p-values (FDR Adj-p) are shown. RA, Relative abundance.

**Phylum**	**Family**	**Max RA**	**Prevalence**	**t**	**P-value**	**FDR Adj-P**
		Day Enriched				
Cyanobacteria	Chroococcales Unclassified	22.396	Common	2.4	1.87E-02	5.18E-02
Gammaproteobacteria	Moraxellaceae	80.626		8.3	1.12E-10	3.50E-08
Cyanobacteria	Pleurocapsales Unclassified	0.262	Rare	2.0	4.72E-02	1.02E-01
	Prochlorotrichaceae	0.055		2.6	1.35E-02	4.10E-02
Firmicutes	Planococcaceae	0.138		2.5	1.48E-02	4.39E-02
	Streptococcaceae	0.065		2.6	1.29E-02	3.97E-02
Betaproteobacteria	Neisseriaceae	0.191		4.4	5.78E-05	3.56E-04
Gammaproteobacteria	Pasteurellaceae	0.331		2.6	1.22E-02	3.84E-02
Verrucomicrobia	Rubritaleaceae	0.251		4.5	5.30E-05	3.33E-04
	Verrucomicrobiaceae	0.158		2.4	2.20E-02	5.86E-02
	Akkermansiaceae	0.127		3.8	4.87E-04	2.25E-03
		Night Enriched				
Alphaproteobacteria	Rhodobacteraceae	63.086	Common	-4.1	1.60E-04	9.11E-04
	Pelagibacteraceae	15.997		-3.7	6.61E-04	3.01E-03
Gammaproteobacteria	Alteromonadaceae	45.653		-4.5	4.09E-05	2.73E-04
	Halomonadaceae	42.258		-3.8	4.61E-04	2.16E-03
Bacteroidetes	Flavobacteriia Unclassified	2.625	Semi-common	-2.8	8.48E-03	2.92E-02
Cyanobacteria	Prochlorococcaceae	3.662		-3.8	4.21E-04	2.03E-03
Alphaproteobacteria	Alphaproteobacteria Unclassified	2.878		-6.8	2.05E-08	9.15E-07
	Bradyrhizobiaceae	0.772		-6.1	2.31E-07	4.83E-06
	Phyllobacteriaceae	0.618		-5.6	1.31E-06	1.79E-05
	Rhizobiaceae	1.106		-5.8	5.36E-07	8.87E-06
	Rhodobacterales Unclassified	1.552		-6.7	2.88E-08	1.00E-06
	Rhodospirillaceae	2.112		-6.8	1.54E-08	8.08E-07
Betaproteobacteria	Burkholderiaceae	0.645		-4.5	5.15E-05	3.30E-04
Deltaproteobacteria	Halobacteriovoraceae	2.986		-3.3	1.70E-03	7.36E-03
Gammaproteobacteria	Cellvibrionaceae	0.771		-5.3	3.23E-06	3.62E-05
	Ectothiorhodospiraceae	0.551		-6.7	2.33E-08	9.15E-07
	Enterobacteriaceae	1.342		-2.4	2.29E-02	6.05E-02
	Halieaceae	0.623		-6.9	1.31E-08	8.08E-07
	Idiomarinaceae	0.908		-2.0	4.91E-02	1.06E-01
	Marinimicrobia - SAR186 (Candidatus)	0.627		-3.3	1.76E-03	7.36E-03
	Oceanospirillaceae	4.345		-4.8	1.87E-05	1.47E-04
	Pseudoalteromonadaceae	1.249		-4.8	1.80E-05	1.45E-04

	Shewanellaceae	0.665		-3.9	2.75E-04	1.44E-03
	Vibrionaceae	1.703		-3.8	3.67E-04	1.83E-03
	Xanthomonadaceae	0.762		-2.7	8.76E-03	2.94E-02
Acidithiobacillia	Acidithiobacillaceae	0.036	Rare	-5.3	3.65E-06	3.82E-05
Acidobacteria	Blastocatellia Unclassified	0.004		-2.4	1.99E-02	5.42E-02
Actinobacteria	Cellulomonadaceae	0.026		-2.6	1.27E-02	3.95E-02
	Corynebacteriaceae	0.131		-2.1	4.53E-02	1.01E-01
	Frankiaceae	0.181		-2.2	3.18E-02	7.67E-02
	Gordoniaceae	0.067		-2.6	1.17E-02	3.75E-02
	Intrasporangiaceae	0.055		-2.7	9.20E-03	3.04E-02
	Kineosporiaceae	0.029		-3.1	3.27E-03	1.27E-02
	Micrococcaceae	0.095		-2.1	4.29E-02	9.83E-02
	Micromonosporaceae	0.183		-2.3	2.66E-02	6.91E-02
	Nocardioidaceae	0.138		-2.3	2.73E-02	7.03E-02
	Nocardiopsaceae	0.143		-2.1	4.36E-02	9.86E-02
	Pseudonocardiaceae	0.168		-2.7	8.81E-03	2.94E-02
	Sporichthyaceae	0.043		-2.4	1.84E-02	5.17E-02
	Streptomycetaceae	0.438		-2.6	1.20E-02	3.81E-02
	Streptosporangiaceae	0.064		-2.9	5.64E-03	2.03E-02
	Thermomonosporaceae	0.050		-2.8	7.67E-03	2.70E-02
	Tsukamurellaceae	0.016		-2.1	4.56E-02	1.01E-01
Aquificae	Aquificaceae	0.046		-2.2	3.32E-02	7.89E-02
	Desulfurobacteriaceae	0.012		-2.3	2.42E-02	6.34E-02
	Hydrogenothermaceae	0.048		-2.2	2.97E-02	7.34E-02
Bacteroidetes	Bacteroidetes Unclassified	0.358		-2.4	2.20E-02	5.86E-02
	Cyclobacteriaceae	0.301		-2.1	3.97E-02	9.23E-02
Chloroflexi	Chloroflexi Unclassified	0.002		-2.0	4.71E-02	1.02E-01
Chrysiogenetes	Chrysiogenaceae	0.022		-3.6	7.66E-04	3.44E-03
Deferribacteres	Deferribacteraceae	0.059		-2.9	5.15E-03	1.88E-02
Deinococcus-Thermus	Deinococcaceae	0.046		-4.1	1.62E-04	9.11E-04
Firmicutes	Alicyclobacillaceae	0.042		-2.4	2.01E-02	5.44E-02
	Leuconostocaceae	0.012		-2.2	2.95E-02	7.34E-02
	Natranaerobiaceae	0.019		-2.8	7.43E-03	2.65E-02
	Paenibacillaceae	0.141		-2.5	1.47E-02	4.39E-02
	Peptococcaceae	0.131		-2.9	4.99E-03	1.86E-02
	Symbiobacteriaceae	0.018		-4.5	4.45E-05	2.91E-04
	Syntrophomonadaceae	0.026		-2.5	1.55E-02	4.44E-02
	Tissierellia Unclassified	0.019		-2.5	1.59E-02	4.51E-02
	Veillonellaceae	0.075		-2.2	3.21E-02	7.69E-02
Fusobacteria	Fusobacteriaceae	0.060		-2.4	1.93E-02	5.33E-02
	Leptotrichiaceae	0.031		-2.1	4.36E-02	9.86E-02
Lentisphaeria	Victivallaceae	0.006		-2.1	4.43E-02	9.93E-02
Nitrospinae	Nitrospinaceae	0.037		-2.9	5.07E-03	1.87E-02

Alphaproteobacteria	Acetobacteraceae	0.483	-6.3	1.03E-07	2.70E-06
	Anaplasmataceae	0.156	-4.7	2.80E-05	2.00E-04
	Aurantimonadaceae	0.272	-5.2	4.60E-06	4.66E-05
	Bartonellaceae	0.164	-5.1	5.53E-06	5.12E-05
	Beijerinckiaceae	0.120	-7.6	1.24E-09	1.30E-07
	Brucellaceae	0.350	-6.1	1.82E-07	4.09E-06
	Hyphomicrobiaceae	0.268	-5.9	4.04E-07	7.05E-06
	Kiloniellaceae	0.262	-7.6	1.22E-09	1.30E-07
	Kordiimonadaceae	0.157	-4.9	1.40E-05	1.19E-04
	Magnetococcaceae	0.056	-3.8	3.73E-04	1.83E-03
	Methylobacteriaceae	0.350	-5.8	5.94E-07	8.88E-06
	Methylocystaceae	0.141	-5.4	2.26E-06	2.83E-05
	Midichloriaceae Candidatus	0.004	-2.5	1.56E-02	4.44E-02
	Paracaedibacteraceae Candidatus	0.027	-4.6	3.32E-05	2.27E-04
	Rhodobiaceae	0.300	-4.8	1.67E-05	1.38E-04
	Rhodospirillales Unclassified	0.020	-4.3	9.75E-05	5.89E-04
	Rickettsiaceae	0.114	-2.6	1.14E-02	3.69E-02
	Xanthobacteraceae	0.242	-5.8	5.68E-07	8.88E-06
Betaproteobacteria	Chromobacteriaceae	0.145	-4.1	1.82E-04	9.92E-04
	Hydrogenophilaceae	0.044	-4.2	1.25E-04	7.40E-04
	Methylophilales Unclassified	0.054	-4.6	3.30E-05	2.27E-04
	Nitrosomonadaceae	0.086	-3.3	2.12E-03	8.65E-03
	Oxalobacteraceae	0.152	-3.1	3.00E-03	1.18E-02
	Rhodocyclaceae	0.193	-6.5	4.42E-08	1.39E-06
	Sulfuricellaceae	0.003	-5.4	2.54E-06	3.06E-05
	Sutterellaceae	0.008	-2.1	4.09E-02	9.44E-02
Deltaproteobacteria	Bdellovibrionaceae	0.179	-4.7	2.26E-05	1.65E-04
	Deltaproteobacteria Unclassified	0.078	-3.4	1.53E-03	6.78E-03
	Desulfobacteraceae	0.108	-2.1	3.97E-02	9.23E-02
	Desulfobulbaceae	0.115	-4.0	2.21E-04	1.18E-03
	Desulfohalobiaceae	0.020	-2.5	1.50E-02	4.39E-02
	Desulfomicrobiaceae	0.020	-3.0	3.80E-03	1.45E-02
	Desulfovibrionaceae	0.134	-3.9	2.92E-04	1.48E-03
	Desulfuromonadaceae	0.113	-3.9	2.89E-04	1.48E-03
	Geobacteraceae	0.193	-3.2	2.74E-03	1.09E-02
	Myxococcaceae	0.060	-2.8	7.91E-03	2.76E-02
	Syntrophaceae	0.029	-3.8	4.35E-04	2.07E-03
	Syntrophobacteraceae	0.024	-2.2	3.07E-02	7.52E-02
Epsilonproteobacteria	Campylobacteraceae	0.097	-2.5	1.43E-02	4.31E-02
	Campylobacterales Unclassified	0.004	-2.3	2.77E-02	7.04E-02

	Epsilonproteobacteria Unclassified	0.021	-2.3	2.78E-02	7.04E-02
	Nautiliaceae	0.016	-2.2	3.17E-02	7.67E-02
Gammaproteobacteria	Aeromonadaceae	0.268	-5.5	1.49E-06	1.95E-05
	Alteromonadales Unclassified	0.065	-4.2	1.28E-04	7.42E-04
	Chromatiaceae	0.266	-5.9	3.89E-07	7.05E-06
	Colwelliaceae	0.172	-5.3	3.37E-06	3.65E-05
	Francisellaceae	0.098	-3.0	3.84E-03	1.45E-02
	Gammaproteobacteria Unclassified	0.361	-6.0	3.14E-07	6.16E-06
	Hahellaceae	0.402	-5.1	5.54E-06	5.12E-05
	Halothiobacillaceae	0.019	-5.7	8.59E-07	1.23E-05
	Methylococcaceae	0.229	-6.4	8.26E-08	2.36E-06
	Microbulbiferaceae	0.123	-5.3	3.12E-06	3.62E-05
	Moritellaceae	0.052	-5.1	7.22E-06	6.48E-05
	Piscirickettsiaceae	0.267	-6.2	1.39E-07	3.37E-06
	Porticoccaceae	0.081	-4.7	2.26E-05	1.65E-04
	Psychromonadaceae	0.093	-4.1	1.83E-04	9.92E-04
	Rhodanobacteraceae	0.061	-4.7	2.11E-05	1.61E-04
	Saccharospirillaceae	0.139	-7.1	6.38E-09	5.01E-07
	Salinisphaeraceae	0.010	-3.3	1.74E-03	7.36E-03
	Sinobacteraceae	0.028	-3.2	2.74E-03	1.09E-02
	Spongiibacteraceae	0.175	-5.0	7.71E-06	6.73E-05
	Succinivibrionaceae	0.011	-2.0	4.73E-02	1.02E-01
	Thiotrichaceae	0.088	-3.3	1.80E-03	7.43E-03
Zetaproteobacteria	Mariprofundaceae	0.028	-5.2	5.15E-06	5.06E-05
	Zetaproteobacteria Unclassified	0.039	-2.3	2.84E-02	7.14E-02
Synergistia	Synergistaceae	0.037	-2.7	8.65E-03	2.94E-02
Thermodesulfobacteria	Thermodesulfobacteriaceae	0.005	-2.5	1.51E-02	4.39E-02
Thermotogae	Fervidobacteriaceae	0.044	-2.7	1.06E-02	3.47E-02
	Kosmotogaceae	0.012	-2.2	3.56E-02	8.41E-02
	Thermotogaceae	0.035	-3.3	1.74E-03	7.36E-03

Supplementary Table 3 | Metabolic pathways enriched during the day and night. Diel enrichment (Cohen's d, the difference in day mean and night mean divided by the standard deviation (Std Dev); |d| > 0.5 is considered moderately enriched) of protein-coding gene families (SEED Level 3 Subsystems). Significant enrichment ($p < 0.05$) were corrected for false discovery rate (FDR Adj-p).

Subsystem Level 1	Subsystem Level 2	Subsystem Level 3	Cohen's d	FDR Adj-p	Mean Day	Mean Night	Std Dev
		**Day Enriched**					
Amino Acids	Amino acid racemase	Amino acid racemase	1.61	4.33E-04	0.0306	0.0328	0.0021
	Arginine; urea cycle, polyamines	Arginine Biosynthesis extended	1.09	1.23E-02	0.0169	0.0211	0.0046
		Polyamine Metabolism	0.02	2.97E-03	0.0349	0.0397	0.0043
		Urea decomposition	0.99	1.64E-02	0.0188	0.0314	0.0096
	Aromatic amino acids & derivatives	Chorismate Synthesis	0.21	3.44E-02	0.0208	0.0329	0.0089
		Common Pathway For Synthesis of Aromatic Compounds (DAHP synthase to chorismate)	0.20	4.34E-02	0.0269	0.0324	0.0042
		Tryptophan synthesis	1.21	4.62E-04	0.0252	0.0139	0.0070
	Branched-chain amino acids	HMG CoA Synthesis	1.23	1.15E-02	0.0598	0.0566	0.0029
	Glutamine, glutamate, aspartate, asparagine; ammonia assimilation	Glutamate dehydrogenases	1.32	3.12E-03	0.0083	0.0121	0.0034
	Histidine Metabolism	Histidine Degradation	1.00	4.27E-03	0.0661	0.0660	0.0049
	Lysine, threonine, methionine, & cysteine	Cysteine Biosynthesis, MCB 432	1.54	2.75E-04	0.0632	0.0548	0.0085
		Lysine fermentation	0.84	2.76E-04	0.0271	0.0365	0.0109
Carbohydrates	Central carbohydrate metabolism	TCA Cycle - Acinetobacter	0.40	7.14E-03	0.0495	0.0486	0.0041
		Glycolysis & Gluconeogenesis	1.29	3.04E-03	0.0450	0.0441	0.0046
		Glyoxylate bypass	0.83	1.34E-02	0.0721	0.0671	0.0042
	Fermentation	Fermentations: Mixed acid	0.91	1.40E-02	0.0402	0.0319	0.0067
	Monosaccharides	Mannose Metabolism	1.01	5.82E-03	0.0303	0.0319	0.0040
	One-carbon Metabolism	Formaldehyde assimilation: Ribulose monophosphate pathway	1.31	1.00E-02	0.0404	0.0468	0.0068
	Organic acids	Methylcitrate cycle	1.17	1.23E-02	0.0146	0.0193	0.0039
Cell Division & Cell Cycle	Bacterial Cytoskeleton	Bacterial Cytoskeleton	1.29	2.28E-03	0.0381	0.0470	0.0071
	Two cell division clusters relating to chromosome partitioning	Two cell division clusters relating to chromosome partitioning	0.87	1.12E-03	0.0401	0.0561	0.0122
Cell Wall & Capsule	Capsular & extracellular polysacchrides	Capsular Polysaccharide (CPS) of Campylobacter	1.01	2.74E-02	0.0350	0.0265	0.0064
	Capsular & extracellular polysacchrides	Capsular Polysaccharides Biosynthesis & Assembly	1.29	2.76E-04	0.0230	0.0299	0.0056
	Capsular & extracellular polysacchrides	Streptococcal Hyaluronic Acid Capsule	1.15	3.08E-02	0.0537	0.0462	0.0075

	Gram-Negative cell wall components	KDO2-Lipid A biosynthesis	1.25	7.81E-05	0.0548	0.0574	0.0052
	Gram-Negative cell wall components	Lipopolysaccharide assembly	0.83	1.79E-02	0.0564	0.0439	0.0081
	Gram-Negative cell wall components	Major Outer Membrane Proteins	1.58	1.57E-04	0.0535	0.0497	0.0044
	Gram-Negative cell wall components	Outer membrane	0.53	2.78E-02	0.0055	0.0081	0.0025
	Murein Hydrolases	Murein Hydrolases	1.25	9.49E-03	0.0429	0.0480	0.0057
	Peptidoglycan biosynthesis--gjo	Peptidoglycan biosynthesis--gjo	0.98	3.70E-02	0.0273	0.0293	0.0019
	Raj MurE	Raj MurE	1.44	7.68E-04	0.0622	0.0656	0.0069
	Recycling of Peptidoglycan Amino Sugars	Recycling of Peptidoglycan Amino Sugars	1.42	4.02E-03	0.0253	0.0331	0.0096
Cofactors, Vitamins, & Pigments	CLO thiaminPP biosynthesis	CLO thiaminPP biosynthesis	1.62	5.61E-04	0.0595	0.0578	0.0043
	Coenzyme A	coA-FolK	1.49	2.76E-04	0.0135	0.0203	0.0051
		dcernst CoA Salvage	1.49	1.52E-03	0.0489	0.0644	0.0131
	Folate & pterines	5-FCL-like protein	1.46	1.97E-03	0.0244	0.0350	0.0102
		Folate Biosynthesis	0.15	6.31E-03	0.0234	0.0326	0.0099
		Pterin carbinolamine dehydratase	1.32	1.56E-03	0.0408	0.0462	0.0046
		Pterin metabolism 3	1.49	1.79E-04	0.0692	0.0614	0.0060
		YgfZ	1.57	2.76E-04	0.0448	0.0387	0.0072
	Lipoic acid	BEY LIP	0.24	8.02E-03	0.0611	0.0679	0.0047
	Molybdopterin cytosine dinucleotide	Molybdopterin cytosine dinucleotide	1.59	1.07E-03	0.0817	0.0875	0.0058
	NAD & NADP	NAD regulation	1.57	3.46E-04	0.0828	0.0833	0.0057
	Quinone cofactors	Pyrroloquinoline Quinone biosynthesis	0.82	3.74E-02	0.0320	0.0421	0.0084
		Ubiquinone Biosynthesis	1.26	4.17E-03	0.0200	0.0276	0.0065
		Ubiquinone biosynthesis -- gjo	1.18	3.05E-03	0.0148	0.0205	0.0049
	Riboflavin, FMN, FAD	Test - Riboflavin	1.38	3.97E-04	0.0266	0.0273	0.0026
	Test - DHFR	Test - DHFR	1.20	1.99E-03	0.0108	0.0158	0.0044
	Test - Thiamin	Test - Thiamin	0.37	4.71E-02	0.0405	0.0547	0.0110
	Thiamin Copy RZ	Thiamin Copy RZ	1.20	2.75E-03	0.0043	0.0080	0.0035
DNA Metabolism	CRISPs	CRISPRs	1.29	1.21E-02	0.0148	0.0227	0.0066
	DNA repair	2-phosphoglycolate salvage	0.88	1.77E-02	0.0050	0.0087	0.0037
		DNA repair, RecBCD pathway	0.94	2.40E-02	0.0136	0.0147	0.0029
		DNA repair, RecFOR pathway	0.50	2.59E-02	0.0294	0.0352	0.0054
		RecA & RecX	1.33	6.52E-03	0.0173	0.0238	0.0051

		Uracil-DNA glycosylase	1.15	3.19E-02	0.0365	0.0311	0.0060
	DNA replication	DNA-replication	1.50	5.61E-04	0.0294	0.0440	0.0102
	Restriction-Modification System	Restriction-Modification System	1.23	4.17E-03	0.0113	0.0209	0.0076
	Type I Restriction-Modification	Type I Restriction-Modification	1.20	5.40E-03	0.0306	0.0389	0.0094
	YcfH	YcfH	1.32	1.10E-02	0.0282	0.0387	0.0089
Dormancy & Sporulation	Persister Cells	Persister Cells	1.01	6.28E-03	0.0038	0.0075	0.0035
Fatty Acids, Lipids, & Isoprenoids	Betaine lipids in bactera	Betaine lipids in bactera	0.40	1.21E-02	0.0335	0.0464	0.0090
	Fatty acids	Fatty acid degradation regulons	1.24	1.21E-02	0.0174	0.0254	0.0082
		Mycobacterial gene cluster associated with resistance against FAS-II antibiotics	1.59	6.76E-04	0.0083	0.0149	0.0049
	Isoprenoids	Isoprenoinds for Quinones	0.94	1.49E-02	0.0181	0.0194	0.0038
		Nonmevalonate Branch of Isoprenoid Biosynthesis	0.17	4.22E-03	0.0429	0.0399	0.0029
	Phospholipids	Glycerolipid & Glycerophospholipid Metabolism in Bacteria	1.22	9.57E-03	0.0149	0.0192	0.0043
	Triacylglycerols	Triacylglycerol metabolism	1.29	9.19E-03	0.0283	0.0393	0.0092
Membrane Transport	Protein & nucleoprotein secretion system, Type IV	Type IV pilus	1.57	2.76E-04	0.0454	0.0408	0.0036
		Twin-arginine translocation system	1.38	3.92E-03	0.0272	0.0284	0.0021
	Transport of Molybdenum	Transport of Molybdenum	1.59	2.75E-04	0.0074	0.0114	0.0043
	Transport of Zinc	Transport of Zinc	1.44	1.97E-03	0.0296	0.0352	0.0042
	TRAP transporters	A TRAP transporter & a hypothetical	1.58	8.71E-04	0.0466	0.0365	0.0086
	Uni- Sym- & Antiporters	Proton-dependent Peptide Transporters	1.49	2.52E-03	0.0195	0.0284	0.0073
Metabolism of Aromatic Compounds	Anaerobic degradation of aromatic compounds	Anaerobic benzoate metabolism	0.27	5.41E-03	0.0051	0.0082	0.0050
	Peripheral pathways for catabolism of aromatic compounds	Chloroaromatic degradation pathway	1.41	1.61E-03	0.0057	0.0166	0.0068
Motility & Chemotaxis	Social motility & nonflagellar swimming in bacteria	Bacterial motility:Gliding	1.46	4.08E-05	0.0077	0.0158	0.0067
Macronutrients	Sulfur Metabolism	Sulfur - Dimethylsulfoniopropionate (DMSP) mineralization, gjo	1.21	5.16E-03	0.0502	0.0657	0.0104
		Sulfur - Dimethylsulfoniopropionate (DMSP) mineralization, WBW	1.21	5.16E-03	0.0375	0.0632	0.0176
		Sulfur Inorganic Sulfur Assimilation	1.45	1.48E-03	0.0133	0.0219	0.0059
		L-Cystine Uptake & Metabolism	1.47	7.68E-04	0.0077	0.0108	0.0030
	Nitrogen Metabolism	Nitrosative stress	1.46	4.53E-04	0.0171	0.0256	0.0060
	Phosphorus Metabolism	Phosphate metabolism	0.31	4.17E-02	0.0627	0.0557	0.0054
	Potassium Metabolism	Potassium homeostasis	1.28	9.19E-03	0.0621	0.0582	0.0044

	Iron acquisition & metabolism	RCJ pfr	0.37	1.63E-03	0.0192	0.0291	0.0063
Phages, Prophages, Transposable elements, Plasmids	Phages, Prophages	Phage tail fiber proteins	0.97	4.56E-02	0.0132	0.0081	0.0051
Protein Metabolism	Protein biosynthesis	Ribosome biogenesis bacterial	0.64	2.81E-03	0.0382	0.0259	0.0095
		Single-copy ribosomal proteins	1.22	9.57E-03	0.0141	0.0179	0.0040
		tRNA aminoacylation, Asp & Asn	1.02	1.85E-02	0.0339	0.0393	0.0052
	Protein degradation	EC 3.4.11.- Aminopeptidases	0.88	7.07E-03	0.0265	0.0329	0.0058
		Proteolysis in bacteria, ATP-dependent	1.13	1.56E-03	0.0469	0.0555	0.0078
	Protein folding	Peptidyl-prolyl cis-trans isomerase	1.21	3.42E-03	0.0413	0.0511	0.0074
		Periplasmic disulfide interchange	1.13	1.47E-02	0.0405	0.0351	0.0047
		Protein chaperones	1.37	8.19E-03	0.0010	0.0049	0.0029
	Protein processing & modification	Lipoprotein Biosynthesis	1.16	1.27E-02	0.0817	0.0744	0.0058
		N-linked Glycosylation in Bacteria	1.42	2.17E-03	0.0100	0.0125	0.0032
		Peptide methionine sulfoxide reductase	1.43	1.25E-03	0.0286	0.0300	0.0042
		Ribosomal protein S12p Asp methylthiotransferase	1.16	1.89E-02	0.0614	0.0506	0.0130
		Signal peptidase	1.43	7.55E-04	0.0342	0.0416	0.0082
Regulation & Cell signaling	cAMP signaling in bacteria	cAMP signaling in bacteria	0.29	4.30E-03	0.0202	0.0340	0.0090
	DNA-binding regulatory proteins, strays	DNA-binding regulatory proteins, strays	0.91	2.15E-02	0.0393	0.0221	0.0109
	Iojap	Iojap	1.47	1.58E-03	0.0230	0.0215	0.0027
	Programmed Cell Death & Toxin-antitoxin Systems	Murein hydrolase regulation & cell death	1.31	2.91E-03	0.0541	0.0488	0.0043
		Toxin-antitoxin replicon stabilization systems	1.38	2.16E-03	0.0391	0.0364	0.0028
	Pseudomonas quinolone signal PQS	Pseudomonas quinolone signal PQS	0.89	3.16E-02	0.0366	0.0303	0.0044
	Two-component regulatory systems in Campylobacter	Two-component regulatory systems in Campylobacter	0.77	1.12E-02	0.0309	0.0259	0.0035
Respiration	Biogenesis of cbb3-type cytochrome c oxidases	Biogenesis of cbb3-type cytochrome c oxidases	1.20	4.30E-02	0.0847	0.0855	0.0059
		Biogenesis of c-type cytochromes	1.48	1.92E-03	0.0216	0.0157	0.0036
	Electron accepting reactions	Ubiquinone Menaquinone-cytochrome c reductase complexes	0.07	7.78E-03	0.0469	0.0405	0.0043
	Electron donating reactions	Na(+)-translocating NADH-quinone oxidoreductase & rnf-like group of electron transport complexes	1.16	7.68E-04	0.0373	0.0382	0.0024
	Quinone oxidoreductase family	Quinone oxidoreductase family	1.24	1.31E-03	0.0390	0.0306	0.0057
	Soluble cytochromes & functionally related electron carriers	Soluble cytochromes & functionally related electron carriers	0.04	1.63E-03	0.0525	0.0409	0.0080

Subsystem Level 1	Subsystem Level 2	Subsystem Level 3	Cohen's d	FDR Adj-p	Mean Day	Mean Night	Std Dev
RNA Metabolism	RNA processing & modification	methyl t6A	1.41	3.14E-03	0.0648	0.0643	0.0032
		RNA processing & degradation, bacterial	0.95	1.02E-02	0.0178	0.0138	0.0030
		RNA pseudouridine syntheses	1.45	3.15E-04	0.0355	0.0284	0.0048
		t(6)A synthesis in bacteria	1.27	9.19E-03	0.0807	0.0613	0.0124
		Threonylcarbamoyladenosine	1.32	2.14E-02	0.0636	0.0625	0.0045
		tRNA processing	0.92	2.38E-02	0.0260	0.0264	0.0022
		YrdC-YciO	1.11	2.95E-03	0.0268	0.0276	0.0022
		YrdC-YciO-Sua5 & associated protein families	1.58	2.02E-04	0.0380	0.0197	0.0115
	Transcription	RNA polymerase III	1.39	4.90E-02	0.0345	0.0187	0.0101
Stress Response	Carbon Starvation	Carbon Starvation	1.17	4.74E-02	0.0427	0.0429	0.0024
	Detoxification	Nudix KE	1.18	8.09E-03	0.0430	0.0434	0.0023
	Flavohaemoglobin	Flavohaemoglobin	1.27	1.21E-02	0.0220	0.0237	0.0044
	Osmotic stress	Osmoregulation	1.48	5.00E-04	0.0255	0.0207	0.0058
		Glutaredoxins	1.23	1.58E-02	0.0688	0.0582	0.0084
		Redox-dependent regulation of nucleus processes	1.10	3.12E-03	0.0417	0.0369	0.0041
Virulence, Disease & Defense	Mycobacterium virulence operon involved in DNA transcription	Mycobacterium virulence operon involved in DNA transcription	0.51	2.52E-03	0.0447	0.0377	0.0050
	Mycobacterium virulence operon possibly involved in quinolinate biosynthesis	Mycobacterium virulence operon possibly involved in quinolinate biosynthesis	0.33	3.45E-02	0.0609	0.0543	0.0054
	Resistance to antibiotics & toxic compounds	Arsenic resistance	1.19	8.45E-03	0.0317	0.0307	0.0028
		BlaR1 Family Regulatory Sensor-transducer Disambiguation	1.46	2.16E-03	0.0307	0.0409	0.0075
		Cobalt-zinc-cadmium resistance	1.18	1.83E-02	0.0465	0.0531	0.0111
		Copper homeostasis	1.27	2.10E-02	0.0295	0.0266	0.0024
		Copper homeostasis: copper tolerance	1.54	1.62E-03	0.0254	0.0142	0.0087
		Methicillin resistance in Staphylococci	1.30	2.47E-04	0.0045	0.0067	0.0029
		Monika MRSA	1.58	2.34E-04	0.0248	0.0222	0.0030
		The mdtABCD multidrug resistance cluster	1.08	7.38E-03	0.0701	0.0739	0.0036
	Bacteriocins, ribosomally synthesized antibacterial peptides	Tolerance to colicin E2	1.48	1.32E-03	0.0136	0.0215	0.0075
	Detection	MLST	1.68	1.09E-04	0.0466	0.0387	0.0084

Night enriched

Amino Acids & Derivatives	Alanine, serine, & glycine	Alanine biosynthesis	-1.03	1.64E-02	0.0483	0.0463	0.0038
		Glycine Biosynthesis	-0.91	3.76E-03	0.0174	0.0109	0.0049
		Glycine cleavage system	-1.10	3.96E-04	0.0287	0.0250	0.0033
		Sarcosine oxidases, monomeric & heterotetrameric	-1.32	2.70E-04	0.1188	0.1069	0.0079
		Sarcosine temp	-1.35	1.86E-04	0.0323	0.0402	0.0115
		Serine Biosynthesis	-1.33	6.76E-04	0.0552	0.0402	0.0122
	Arginine; urea cycle, polyamines	Arginine Deiminase Pathway	-1.11	6.37E-03	0.0465	0.0349	0.0097
	Aromatic amino acids & derivatives	Aromatic amino acid interconversions with aryl acids	-0.86	1.79E-04	0.0271	0.0230	0.0032
	Branched-chain amino acids	HMG-CoA	-0.40	6.31E-03	0.0231	0.0198	0.0033
		Isoleucine degradation	-0.95	7.14E-03	0.0046	0.0089	0.0042
		Ketoisovalerate oxidoreductase	-1.22	7.03E-03	0.0088	0.0167	0.0056
		Leucine Degradation & HMG-CoA Metabolism	-1.26	2.39E-02	0.0055	0.0044	0.0029
	Creatine & Creatinine Degradation	Creatine & Creatinine Degradation	-1.31	7.95E-05	0.0556	0.0428	0.0103
	Glutamine, glutamate, aspartate, asparagine; ammonia assimilation	Glutamine synthetases	-1.24	2.76E-04	0.0134	0.0023	0.0070
	Lysine, threonine, methionine, & cysteine	Cysteine Biosynthesis	-0.50	1.61E-04	0.0092	0.0129	0.0066
		Threonine anaerobic catabolism gene cluster	-1.00	1.82E-02	0.0063	0.0117	0.0043
		Threonine degradation	-0.90	2.34E-03	0.0414	0.0434	0.0025
	Proline & 4-hydroxyproline	Proline Synthesis	-1.05	2.14E-02	0.0114	0.0131	0.0018
		Proline, 4-hydroxyproline uptake & utilization	-0.48	1.59E-03	0.0329	0.0296	0.0035
Carbohydrates	Aminosugars	Chitin & N-acetylglucosamine utilization	-0.81	2.00E-02	0.0348	0.0345	0.0020
	Central carbohydrate metabolism	Dihydroxyacetone kinases	-1.33	2.69E-02	0.0852	0.0804	0.0039
		Entner-Doudoroff Pathway	-1.18	2.76E-04	0.0478	0.0540	0.0062
		Ethylmalonyl-CoA pathway of C2 assimilation	-1.05	7.95E-05	0.0340	0.0226	0.0088
		Ethylmalonyl-CoA pathway of C2 assimilation, GJO	-0.94	1.78E-04	0.0300	0.0287	0.0033

	Glycolate, glyoxylate interconversions	-1.19	2.45E-02	0.0143	0.0181	0.0076
	Pyruvate metabolism I: anaplerotic reactions, PEP	-1.44	7.32E-04	0.0119	0.0189	0.0063
	Pyruvate metabolism II: acetyl-CoA, acetogenesis from pyruvate	-1.01	2.14E-02	0.0083	0.0133	0.0046
	TCA Cycle	-0.09	3.77E-02	0.0160	0.0086	0.0051
CO2 fixation	Calvin-Benson cycle	-1.19	4.36E-03	0.0277	0.0383	0.0102
Di- & oligosaccharides	Beta-Glucoside Metabolism	-1.17	2.76E-04	0.1074	0.1063	0.0036
	Fructooligosaccharides(FOS) & Raffinose Utilization	-1.18	9.19E-03	0.0031	0.0076	0.0036
	Lactose & Galactose Uptake & Utilization	-0.29	4.77E-02	0.0499	0.0578	0.0080
	Lactose utilization	-1.14	1.54E-03	0.0169	0.0253	0.0097
	Maltose & Maltodextrin Utilization	-1.29	4.97E-03	0.0871	0.0784	0.0068
	Melibiose Utilization	-1.06	7.64E-03	0.0262	0.0333	0.0047
	Sucrose utilization	-1.20	2.52E-06	0.0185	0.0316	0.0099
	Sucrose utilization Shewanella	-1.00	4.70E-03	0.0060	0.0090	0.0025
	Trehalose Uptake & Utilization	-0.39	4.97E-02	0.0468	0.0402	0.0054
Fermentation	Acetoin, butanediol metabolism	-1.09	4.24E-02	0.0468	0.0402	0.0054
	Fermentations: Lactate	-1.27	9.30E-04	0.0481	0.0416	0.0045
Monosaccharides	Deoxyribose & Deoxynucleoside Catabolism	-1.43	2.76E-04	0.0494	0.0538	0.0042
	D-galactonate catabolism	-1.27	7.95E-05	0.0157	0.0063	0.0064
	D-Galacturonate & D-Glucuronate Utilization	-0.89	9.43E-03	0.0166	0.0295	0.0092
	D-ribose utilization	-1.17	6.87E-03	0.0290	0.0416	0.0101
	D-Sorbitol(D-Glucitol) & L-Sorbose Utilization	-1.03	1.77E-02	0.0441	0.0665	0.0172
	Fructose utilization	-1.43	2.76E-04	0.0555	0.0620	0.0097
	L-Arabinose utilization	-0.98	4.17E-02	0.0504	0.0554	0.0088
	L-fucose utilization temp	-1.34	5.12E-03	0.0154	0.0222	0.0066
	L-rhamnose utilization	-0.34	3.31E-02	0.0041	0.0063	0.0036

		Unspecified monosaccharide transport cluster	-0.98	1.08E-02	0.0209	0.0324	0.0107
		Xylose utilization	-1.20	5.00E-04	0.0075	0.0136	0.0052
	One-carbon Metabolism	One-carbon metabolism by tetrahydropterines	-0.57	1.32E-02	0.0109	0.0241	0.0101
	Organic acids	2-methylcitrate to 2-methylaconitate metabolism cluster	-0.94	2.13E-02	0.0180	0.0266	0.0070
		Glycerate metabolism	-1.33	7.53E-04	0.0648	0.0278	0.0236
		Propionyl-CoA to Succinyl-CoA Module	-1.21	7.95E-05	0.0233	0.0316	0.0082
		Tricarballylate Utilization	-0.63	2.76E-04	0.0385	0.0499	0.0163
	Sugar alcohols	Erythritol utilization	-1.61	5.81E-04	0.0081	0.0140	0.0055
		Ethanolamine utilization	-1.21	2.21E-02	0.0272	0.0225	0.0034
		Glycerol & Glycerol-3-phosphate Uptake & Utilization	-1.50	8.02E-03	0.1098	0.1295	0.0208
		Inositol catabolism	-1.46	5.61E-04	0.0231	0.0300	0.0075
		Mannitol Utilization	-1.46	7.06E-04	0.0134	0.0056	0.0049
	Unknown carbohydrate utilization (cluster Ydj)	Unknown carbohydrate utilization (cluster Ydj)	-1.44	2.10E-03	0.0075	0.0125	0.0038
Cell Wall & Capsule	Capsular & extracellular polysacchrides	Capsular heptose biosynthesis	-1.55	3.15E-04	0.0280	0.0109	0.0109
		Colanic acid biosynthesis	-0.97	2.76E-04	0.0264	0.0398	0.0093
		dTDP-rhamnose synthesis	-1.06	2.88E-02	0.0285	0.0216	0.0047
		Exopolysaccharide Biosynthesis	-1.11	2.16E-03	0.0232	0.0225	0.0028
		Rhamnose containing glycans	-1.10	7.68E-04	0.0169	0.0213	0.0051
		Sialic Acid Metabolism	-1.32	2.25E-03	0.0143	0.0229	0.0063
		YjbEFGH Locus Involved in Exopolysaccharide Production	-1.34	3.54E-03	0.0072	0.0115	0.0046
	Gram-Negative cell wall components	Lipid A modifications	-0.77	9.93E-03	0.0243	0.0291	0.0047
		Lipid A-Ara4N pathway (Polymyxin resistance)	-0.33	2.80E-02	0.0151	0.0242	0.0074
		Lipopolysaccharide-related cluster in Alphaproteobacteria	-0.90	1.32E-03	0.0352	0.0385	0.0055
		LOS core oligosaccharide biosynthesis	-1.53	4.62E-04	0.0158	0.0232	0.0068
Cofactors,	Biotin	Biotin synthesis & utilization	-0.14	1.94E-02	0.0233	0.0307	0.0061

Vitamins, & Pigments	Coenzyme A	Coenzyme A Biosynthesis	-0.37	9.76E-03	0.0280	0.0318	0.0043
	Lipoic acid	Lipoate transport	-0.18	2.49E-03	0.0068	0.0129	0.0058
		Lipoic acid metabolism	-0.38	4.56E-02	0.0162	0.0108	0.0038
	Pyridoxine	Pyridoxin (Vitamin B6) Biosynthesis	-0.06	1.89E-02	0.0047	0.0075	0.0030
		Test Pyridoxin B6	-0.15	9.57E-03	0.0092	0.0144	0.0040
	Quinone cofactors	Menaquinone biosynthesis from chorismate via 1,4-dihydroxy-2-naphthoate	-0.37	9.25E-03	0.0075	0.0107	0.0032
	Tetrapyrroles	Cobalamin synthesis	-1.37	6.04E-04	0.0056	0.0082	0.0038
		Coenzyme B12 biosynthesis	-0.60	3.84E-02	0.0128	0.0170	0.0055
DNA Metabolism	DNA phosphorothioation	DNA phosphorothioation	-0.78	3.16E-02	0.0444	0.0614	0.0124
	DNA repair	DNA Repair Base Excision	-1.04	2.74E-02	0.0552	0.0889	0.0218
		DNA repair, bacterial DinG & relatives	-1.05	1.18E-02	0.0161	0.0239	0.0063
	DNA replication	Plasmid replication	-0.69	3.56E-04	0.0642	0.0456	0.0127
Dormancy & Sporulation	Spore Core Dehydration	Spore Core Dehydration	-1.04	9.43E-04	0.0068	0.0087	0.0021
	Sporulation gene orphans	Sporulation gene orphans	-1.41	5.00E-04	0.0387	0.0480	0.0068
Fatty Acids, Lipids, & Isoprenoids	Head-to-head olefinic hydrocarbon biosynthesis	Head-to-head olefinic hydrocarbon biosynthesis	-0.56	3.34E-03	0.0075	0.0107	0.0034
	Isoprenoids	Acyclic terpenes utilization	-1.28	2.41E-02	0.0839	0.0884	0.0048
		Isoprenoid Biosynthesis	-0.81	3.20E-02	0.0531	0.0587	0.0069
		Isoprenoid Biosynthesis: Interconversions	-0.96	4.55E-03	0.0565	0.0602	0.0033
	Polyhydroxybutyrate metabolism	Polyhydroxybutyrate metabolism	-1.00	1.89E-02	0.0356	0.0404	0.0061
Membrane Transport	ABC transporters	ABC transporter alkylphosphonate	-1.25	1.44E-03	0.0063	0.0106	0.0035
		ABC transporter branched-chain amino acid	-1.31	4.62E-04	0.0157	0.0203	0.0039
		ABC transporter dipeptide	-0.67	3.15E-03	0.0137	0.0193	0.0055
		ABC transporter oligopeptide	-0.57	1.21E-02	0.0147	0.0195	0.0040
		ABC transporter tungstate	-1.03	6.19E-04	0.0098	0.0065	0.0033
		ATP-dependent efflux pump transporter Ybh	-0.63	4.17E-02	0.0150	0.0206	0.0055

		Periplasmic-Binding-Protein-Dependent Transport System for Glucosides	-1.07	2.76E-04	0.0020	0.0048	0.0035
	ECF class transporters	ECF class transporters	-1.17	4.83E-05	0.0033	0.0077	0.0039
	Protein & nucleoprotein secretion system, Type IV	Mannose-sensitive hemagglutinin type 4 pilus	-1.31	1.79E-04	0.0592	0.0571	0.0033
		pVir Plasmid of Campylobacter	-1.22	4.48E-03	0.0766	0.0708	0.0047
	Protein secretion system, Type II	General Secretion Pathway	-1.01	4.97E-03	0.0234	0.0262	0.0031
		Widespread colonization island	-0.70	1.63E-03	0.0397	0.0356	0.0040
	Protein secretion system, Type VIII (Extracellular nucleation/precipitation pathway, ENP)	Curli production	-1.07	4.17E-03	0.0060	0.0103	0.0041
	Ton & Tol transport systems	Ton & Tol transport systems	-0.94	9.86E-04	0.0562	0.0534	0.0032
	Transport of Manganese	Transport of Manganese	-0.92	3.56E-03	0.0118	0.0164	0.0050
	Transport of Nickel & Cobalt	Transport of Nickel & Cobalt	-1.30	7.64E-03	0.0163	0.0209	0.0054
	TRAP transporters	Tricarboxylate transport system	-1.44	1.24E-02	0.0306	0.0462	0.0104
Metabolism of Aromatic Compounds	Aromatic Amin Catabolism	Aromatic Amin Catabolism	-0.87	1.73E-02	0.0832	0.0769	0.0056
	Benzoate transport & degradation cluster	Benzoate transport & degradation cluster	-1.37	1.38E-03	0.0204	0.0277	0.0057
	Metabolism of central aromatic intermediates	4-Hydroxyphenylacetic acid catabolic pathway	-0.93	7.83E-03	0.0387	0.0307	0.0066
		Catechol branch of beta-ketoadipate pathway	-1.01	1.35E-03	0.0402	0.0334	0.0060
		Central meta-cleavage pathway of aromatic compound degradation	-1.23	7.87E-03	0.0572	0.0504	0.0050
		Homogentisate pathway of aromatic compound degradation	-0.59	1.89E-02	0.0322	0.0283	0.0034
		N-heterocyclic aromatic compound degradation	-1.08	3.96E-04	0.0426	0.0314	0.0079
		Protocatechuate branch of beta-ketoadipate pathway	-1.22	9.53E-03	0.0400	0.0347	0.0037
		Salicylate & gentisate catabolism	-0.88	2.91E-03	0.0288	0.0261	0.0023
	Peripheral pathways for catabolism of aromatic compounds	Biphenyl Degradation	-1.05	2.71E-03	0.0131	0.0148	0.0018
		Naphtalene & antracene degradation	-0.95	1.21E-02	0.0287	0.0243	0.0031
		p-Hydroxybenzoate degradation	-1.30	1.03E-02	0.0048	0.0090	0.0038

		Salicylate ester degradation	-1.00	5.12E-03	0.0158	0.0212	0.0047
		Toluene degradation	-0.69	4.39E-04	0.0654	0.0636	0.0062
	Phenylacetyl-CoA catabolic pathway (core)	Phenylacetyl-CoA catabolic pathway (core)	-0.76	3.56E-02	0.0270	0.0218	0.0058
Motility & Chemotaxis	Flagellar motility in Prokaryota	Flagellar motility	-1.37	9.78E-04	0.0132	0.0210	0.0055
		Flagellum	-1.55	5.81E-04	0.0326	0.0230	0.0065
		Flagellum in Campylobacter	-1.25	5.46E-03	0.0376	0.0256	0.0092
Macronutrients	Nitrogen Metabolism	Cyanate hydrolysis	-1.10	2.62E-03	0.0087	0.0150	0.0042
		Dissimilatory nitrite reductase	-1.11	4.16E-02	0.0121	0.0139	0.0041
	Sulfur Metabolism	Bacterial pathways for dimethylsulfoniopropionate & acrylate catabolism	-1.52	1.84E-04	0.0111	0.0050	0.0044
		CFE Sulfur Oxidation	-1.32	1.24E-04	0.0135	0.0097	0.0043
		Dimethylsulfoniopropionate (DMSP) mineralization	-1.18	7.80E-04	0.0042	0.0098	0.0051
		Organic sulfur assimilation - CsdL protein family	-1.06	1.58E-03	0.0233	0.0208	0.0032
		Sulfur oxidation	-1.41	7.95E-05	0.0290	0.0400	0.0084
	Phosphorus Metabolism	Phosphonate metabolism	-1.24	1.38E-03	0.0454	0.0369	0.0070
		Alkylphosphonate utilization	-1.04	1.83E-02	0.0061	0.0121	0.0050
	Potassium Metabolism	pH adaptation potassium efflux system	-0.87	2.76E-04	0.0027	0.0044	0.0034
		Glutathione-regulated potassium-efflux system & associated functions	-1.00	2.16E-03	0.0219	0.0313	0.0090
	Iron acquisition & Metabolism	Siderophore Aerobactin	-0.49	3.34E-02	0.0794	0.0676	0.0080
Nucleosides & Nucleotides	Adenosyl nucleosidases	Adenosyl nucleosidases	-0.90	3.96E-02	0.0113	0.0177	0.0055
	Hydantoin metabolism	Hydantoin metabolism	-1.36	1.05E-02	0.0501	0.0571	0.0059
	Pseudouridine Metabolism	Pseudouridine Metabolism	-0.96	2.76E-04	0.0727	0.0737	0.0034
	Purines	Purine conversions	-0.94	3.01E-02	0.0077	0.0115	0.0044
		Purine Utilization	-0.81	3.00E-02	0.0421	0.0419	0.0031
	Pyrimidines	pyrimidine conversions	-1.12	2.95E-02	0.0216	0.0333	0.0095
		Pyrimidine utilization	-0.78	2.16E-03	0.0116	0.0218	0.0105
Phages,	Integrons	Integrons	-1.22	6.49E-03	0.0643	0.0522	0.0104

Prophages, Transposons, & Plasmids	Pathogenicity islands	Staphylococcal pathogenicity islands SaPI	-1.19	4.06E-03	0.0499	0.0573	0.0058
	Phages, Prophages	Phage head & packaging	-1.02	1.89E-02	0.0305	0.0250	0.0045
		Phage packaging machinery	-1.21	1.30E-02	0.0050	0.0093	0.0030
		Staphylococcal phi-Mu50B-like prophages	-1.03	3.85E-03	0.0406	0.0404	0.0042
	Plasmid related functions	Plasmid-encoded T-DNA transfer	-0.79	9.15E-03	0.0119	0.0061	0.0041
Photosynthesis	Electron transport & photophosphorylation	Photosystem II-type photosynthetic reaction center	-1.12	2.94E-03	0.0564	0.0523	0.0043
Protein Metabolism	Protein biosynthesis	Translation elongation factor G family	-0.91	1.73E-03	0.0417	0.0298	0.0082
		tRNA aminoacylation, His	-1.05	5.41E-03	0.0122	0.0101	0.0016
	Protein degradation	EC 3.4.19.- Omega peptidases	-0.91	7.64E-03	0.0128	0.0103	0.0019
		EC 3.4.21.- Serine endopeptidase	-0.86	1.24E-02	0.0542	0.0492	0.0055
		Proteasome bacterial	-1.50	4.80E-04	0.0332	0.0270	0.0056
		Putative TldE-TldD proteolytic complex	-1.27	5.61E-04	0.0471	0.0361	0.0070
	Protein processing & modification	Ribosomal protein S5p acylation	-0.98	2.41E-02	0.0060	0.0006	0.0039
	Secretion	Protein secretion by ABC-type exporters	-1.12	3.02E-03	0.0050	0.0076	0.0025
	Selenoproteins	Selenocysteine metabolism	-1.14	5.41E-03	0.0026	0.0048	0.0029
Regulation & Cell signaling	Global Two-component Regulator PrrBA in Proteobacteria	Global Two-component Regulator PrrBA in Proteobacteria	-1.41	3.08E-03	0.0026	0.0047	0.0028
	Programmed Cell Death & Toxin-antitoxin Systems	Phd-Doc, YdcE-YdcD toxin-antitoxin (programmed cell death) systems	-1.20	6.71E-03	0.0293	0.0494	0.0150
	Rcs phosphorelay signal transduction pathway	Rcs phosphorelay signal transduction pathway	-1.49	7.09E-04	0.0322	0.0219	0.0088
	Regulation of virulence	A conserved operon linked to TyrR & possibly involved in virulence	-1.10	1.97E-02	0.0181	0.0238	0.0039
	Sex pheromones in Enterococcus faecalis & other Firmicutes	Sex pheromones in Enterococcus faecalis & other Firmicutes	-0.44	3.55E-02	0.0282	0.0238	0.0037
	The Chv regulatory system of Alphaproteobacteria	The Chv regulatory system of Alphaproteobacteria	-1.04	7.68E-04	0.0227	0.0345	0.0076

Respiration	ATP synthases	V-Type ATP synthase	-0.49	7.07E-03	0.0092	0.0134	0.0041
	Biogenesis of cytochrome c oxidases	Biogenesis of cytochrome c oxidases	-1.32	2.47E-04	0.0466	0.0400	0.0051
	Carbon monoxide dehydrogenase maturation factors	Carbon monoxide dehydrogenase maturation factors	-1.14	1.81E-04	0.0320	0.0406	0.0056
	Electron accepting reactions	Anaerobic respiratory reductases	-1.19	7.72E-05	0.0164	0.0228	0.0080
		Terminal cytochrome C oxidases	-0.31	1.03E-02	0.0295	0.0188	0.0073
		Terminal cytochrome O ubiquinol oxidase	-0.88	1.94E-02	0.0184	0.0286	0.0081
	Electron donating reactions	CO Dehydrogenase	-1.23	1.84E-04	0.0482	0.0432	0.0041
		Hydrogenases	-0.98	2.15E-02	0.0516	0.0537	0.0036
	Formate hydrogenase	Formate hydrogenase	-1.29	1.77E-02	0.0443	0.0476	0.0039
	Sodium Ion-Coupled Energetics	Na+ translocating decarboxylases & related biotin-dependent enzymes	-1.45	1.58E-03	0.0053	0.0097	0.0042
Secondary Metabolism	Bacterial cytostatics, differentiation factors & antibiotics	Phenazine biosynthesis	-1.04	2.18E-02	0.0216	0.0171	0.0041
	Biosynthesis of phenylpropanoids	Biflavanoid biosynthesis	-0.75	9.15E-03	0.0167	0.0186	0.0022
		Tannin biosynthesis	-0.73	1.07E-02	0.0366	0.0398	0.0028
Stress Response	Bacterial hemoglobins	Bacterial hemoglobins	-1.34	1.81E-03	0.0138	0.0172	0.0053
	Detoxification	Housecleaning nucleoside triphosphate pyrophosphatases	-1.45	1.24E-04	0.0210	0.0293	0.0070
		Uptake of selenate & selenite	-1.57	7.95E-05	0.0507	0.0483	0.0049
	Dimethylarginine metabolism	Dimethylarginine metabolism	-1.01	1.32E-02	0.0186	0.0175	0.0031
	Hfl operon	Hfl operon	-1.54	6.37E-03	0.0367	0.0307	0.0050
	Osmotic stress	Ectoine biosynthesis & regulation	-0.80	6.31E-03	0.0387	0.0290	0.0067
		Synthesis of osmoregulated periplasmic glucans	-1.27	2.70E-04	0.1230	0.1035	0.0165
		Glutathione: Biosynthesis & gamma-glutamyl cycle	-0.60	1.48E-03	0.1097	0.0899	0.0156
		Glutathione: Non-redox reactions	-0.86	2.41E-02	0.0349	0.0191	0.0102
		Glutathionylspermidine & Trypanothione	-1.07	2.82E-02	0.0201	0.0161	0.0030
		Rubrerythrin	-0.92	2.74E-02	0.0175	0.0102	0.0046

	Periplasmic Stress	Periplasmic Stress Response	-1.13	2.55E-02	0.0096	0.0057	0.0036
	Phage shock protein (psp) operon	Phage shock protein (psp) operon	-0.63	2.16E-03	0.0120	0.0178	0.0044
	SigmaB stress responce regulation	SigmaB stress responce regulation	-1.19	1.86E-04	0.0529	0.0590	0.0242
Virulence, Disease & Defense	Resistance to antibiotics & toxic compounds	Zinc resistance	-1.33	3.38E-02	0.0193	0.0078	0.0078
	Type III, Type IV, Type VI, ESAT secretion systems	Type 4 secretion & conjugative transfer	-0.25	4.91E-02	0.0272	0.0093	0.0107
	Invasion & intracellular resistance	Cytolysin & Lipase operon in Vibrio	-0.89	3.01E-02	0.0013	0.0035	0.0024
