# Supplementary material for: Diel population and functional synchrony of microbial communities on coral reefs
Source: Nat Commun. 2019 Apr 12;10:1691. doi: 10.1038/s41467-019-09419-z (PMC6461649; doi:10.1038/s41467-019-09419-z)
Supplement: Supplementary file 3 — Reporting Summary [file 41467_2019_9419_MOESM3_ESM.pdf]

nature research

Corresponding author(s): Linda Wegley Kelly
NCOMMS-18-13952C

Last updated by author(s): Mar 11, 2019

Reporting Summary

Nature Research wishes to improve the reproducibility of the work that we publish. This form provides structure for consistency and transparency in reporting. For further information on Nature Research policies, see Authors & Referees and the Editorial Policy Checklist.

Statistics

For all statistical analyses, confirm that the following items are present in the figure legend, table legend, main text, or Methods section.

n/a	Confirmed	
☐	☒	The exact sample size (*n*) for each experimental group/condition, given as a discrete number and unit of measurement
☐	☒	A statement on whether measurements were taken from distinct samples or whether the same sample was measured repeatedly
☐	☒	The statistical test(s) used AND whether they are one- or two-sided<br>*Only common tests should be described solely by name; describe more complex techniques in the Methods section.*
☒	☐	A description of all covariates tested
☐	☒	A description of any assumptions or corrections, such as tests of normality and adjustment for multiple comparisons
☐	☒	A full description of the statistical parameters including central tendency (e.g. means) or other basic estimates (e.g. regression coefficient) AND variation (e.g. standard deviation) or associated estimates of uncertainty (e.g. confidence intervals)
☐	☒	For null hypothesis testing, the test statistic (e.g. *F*, *t*, *r*) with confidence intervals, effect sizes, degrees of freedom and *P* value noted<br>*Give P values as exact values whenever suitable.*
☒	☐	For Bayesian analysis, information on the choice of priors and Markov chain Monte Carlo settings
☐	☒	For hierarchical and complex designs, identification of the appropriate level for tests and full reporting of outcomes
☐	☒	Estimates of effect sizes (e.g. Cohen's *d*, Pearson's *r*), indicating how they were calculated

Our web collection on statistics for biologists contains articles on many of the points above.

Software and code

Policy information about availability of computer code

Data collection	No software was used.
Data analysis	Sequence reads were compared to the SEED database for metabolic and taxonomic assignments using SUPERFOCUS, which aligns sequence similarities using RAPSearch2 and performs a 98% clustering of the proteins in the database to reduce computational taxation. Metagenomic reads aligned against the SEED database to get the functional annotation were subsequently extracted to identify the taxa that are encoding the respective protein coding genes from NCBI using Taxonkit (http://bioinf.shenwei.me/taxonkit/). Shotgun sequence metagenomic libraries generated ~17 million reads with an average length of 225 base pairs after low quality reads were removed using Prinseq. Community dissimilarity was derived from Bray-Curtis distances calculated in R using the Vegan Package. Putative SSU rRNA gene sequences (25,316) were extracted from the shotgun libraries using GenomePeek and aligned to the SILVA v115 SSU database. Alignment, classification, sequence distance calculation, OTU clustering, phylogenetic tree construction and calculation of among-sample phylogenetic distances were done using the software package mothur. Statistical analyses were completed in R using the Vegan Package and with JMP Pro v13 (SAS, Cary NC, USA).

For manuscripts utilizing custom algorithms or software that are central to the research but not yet described in published literature, software must be made available to editors/reviewers. We strongly encourage code deposition in a community repository (e.g. GitHub). See the Nature Research guidelines for submitting code & software for further information.

Data

Policy information about availability of data

All manuscripts must include a data availability statement. This statement should provide the following information, where applicable:

- Accession codes, unique identifiers, or web links for publicly available datasets
- A list of figures that have associated raw data
- A description of any restrictions on data availability

The biochemical (e.g., DOC, oxygen, pH) data that support the findings of this study have been

deposited in [BCO-DMO (https://www.bco-dmo.org/project/675025)] under the dataset collection DIEL_REEFS and the metagenomic sequence data has been deposited into the [SRA] under accession codes SAMN10442328-SAMN10442375with the following project code [NCBI, https://www.ncbi.nlm.nih.gov/bioproject/504905]. The source data underlying Figures 2, 3, S2 and Tables S2, S3 are provided as a Source Data file.

Field-specific reporting

Please select the one below that is the best fit for your research. If you are not sure, read the appropriate sections before making your selection.

☐ Life sciences ☐ Behavioural & social sciences ☒ Ecological, evolutionary & environmental sciences

For a reference copy of the document with all sections, see nature.com/documents/nr-reporting-summary-flat.pdf

Ecological, evolutionary & environmental sciences study design

All studies must disclose on these points even when the disclosure is negative.

Study description	On each of the four islands, samples were collected at one reef site without a benthic chamber at t12 to represent an open nighttime reef community without confinement. Time-zero samples were collected immediately after benthic chambers were deployed and therefore should be representative of the ambient reef water community.
Research sample	Reef water bacterioplankton samples<br>Discrete water samples for chemical analyses
Sampling strategy	Discrete water samples were collected from each reef plot (N=16) over a 24-hour diel cycle: between 0900-1159 on day 1 (t0), 2100-2359 on day 1 (t12) and 0900-1159 on day 2 (t24) for a total of 48 samples. Reef water samples (1 to 3 liters) were pumped through 0.22 µm 47mm polyethersulfone filters (Sterivex, EMD Millipore, Billerica MA, USA) that were subsequently dried and frozen at -20 degrees C.
Data collection	Data comes from sequencing, analytical analysis of carbon, and autonomous loggers that measure temperature, oxygen, pH, and conductivity.
Timing and spatial scale	The data presented here were collected on a cruise to the southern Line Islands located in the Republic of Kiribati (Central Pacific) from October 22 through November 6, 2013. The study site encompasses three uninhabited coral islands and one atoll: Vostok (-10.0609, -152.309), Millennium (-9.95080, -150.215), Starbuck (-5.62891, -155.925), and Malden (-4.01407, -154.973) separated by 800km of latitudinal distance and exhibiting variance in ocean productivity and nutrient regimes between islands (29). On each island, seawater overlying the benthos (< 0.5m from the bottom) was collected from four distinct forereef sites over a 24-hour period (N=16).
Data exclusions	No data were excluded from the analyses
Reproducibility	Four replicate reef sites were randomly chosen per island and the study included four different island locations. This strategy provided statistical power and reproducibility.
Randomization	Reef sites were chosen by stratified random process.
Blinding	na

Did the study involve field work? ☒ Yes ☐ No

Field work, collection and transport

Field conditions	Tropical marine waters
Location	Coral reefs in the central Pacific at ten meters depth.
Access and import/export	This work was carried out under research permits from the Environment and Conservation Division of the Republic of Kiribati.
Disturbance	Sampling was non-destructive.

Reporting for specific materials, systems and methods

We require information from authors about some types of materials, experimental systems and methods used in many studies. Here, indicate whether each material, system or method listed is relevant to your study. If you are not sure if a list item applies to your research, read the appropriate section before selecting a response.

Materials & experimental systems

n/a	Involved in the study
☒	☐ Antibodies
☒	☐ Eukaryotic cell lines
☒	☐ Palaeontology
☒	☐ Animals and other organisms
☒	☐ Human research participants
☒	☐ Clinical data

Methods

n/a	Involved in the study
☒	☐ ChIP-seq
☒	☐ Flow cytometry
☒	☐ MRI-based neuroimaging
